# Rethinking Matrigel: The Complex Journey to Matrix Alternatives in Organoid Culture

**DOI:** 10.1002/advs.202508734

**Published:** 2025-11-07

**Authors:** Lisa Wolff, Sven Hendrix

**Affiliations:** ^1^ Institute for Translational Medicine Medical School Hamburg Am Kaiserkai 1 20457 Hamburg Germany

**Keywords:** 3D cell culture, extracellular matrix, matrix, organoid, translational models

## Abstract

Matrigel is a crucial tool in cell biology, particularly for organoid research. It forms a stable scaffold with components like basement membrane proteins and growth factors, resembling the extracellular matrix and mimicking the cellular microenvironment. Its complex composition is both an asset and a drawback, as it is undefined and can vary from batch to batch. Another issue is the murine origin of Matrigel, raising ethical and scientific concerns. Interspecies variation hinders the successful translation of research findings from experimental models to clinical application in humans. Despite these well‐known and often‐discussed disadvantages, Matrigel is often the first choice of matrix. This review explores why Matrigel remains the gold standard for many human 3D culture systems despite its murine origin and well‐known limitations. Therefore, challenges are identified that prevent researchers from transitioning to matrix alternatives and, eventually, developing completely xeno‐free human model systems. To tackle these challenges, it is suggested to move beyond a one‐for‐all approach by pursuing a tissue‐ and model‐specific focus when designing new matrices or selecting an alternative from already available options. To facilitate the implementation of Matrigel substitutes, we provide a matrix selection checklist and a scaffold assessment tool, and quantitative assessment criteria to evaluate matrix‐model compatibility are suggested.

## Graphical abstract

Matrigel remains the most widely used scaffold in organoid and 3D culture despite its murine origin, variability, and translational limitations. This review discusses the barriers to replacing Matrigel with xeno‐free alternatives and introduces practical tools, including a matrix selection checklist and scaffold assessment criteria, to guide the development and selection of tissue‐ and model‐specific matrices.

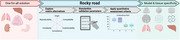

## 1 Introduction

Organoids have become a groundbreaking technology in developmental biology, disease modeling, and regenerative medicine.^[^ ^1^, ^2^, ^3^ ^]^ Derived from stem cells or tissue‐specific progenitor cells, organoids are self‐organizing structures that mimic the architecture and functionality of specific tissues or organs. By recapitulating morphological and functional aspects of their in vivo counterparts, organoids offer valuable insights into cell differentiation, tissue morphogenesis, and intercellular interactions.^[^ ^4^ ^]^ Cerebral organoids from human induced pluripotent stem cells (iPS), for example, form the specific layers of the human cortex and are electrophysiologically active.^[^ ^5^, ^6^ ^]^

Many organoid protocols rely on using a supportive component that mimics the extracellular matrix (ECM), a noncellular scaffold present in native tissues. Its structural and physical properties arise from fibrous proteins, like elastins and laminins, along with proteoglycans and glycosaminoglycans, which form a network that retains water, confers elasticity, and resists compressive forces.^[^ ^7^, ^8^ ^]^ Beyond providing structural support, the ECM plays a vital role in morphogenesis, homeostasis, and differentiation by supplying biochemical and mechanical cues through binding of growth factors and interaction with cellular receptors.^[^ ^7^, ^9^, ^10^ ^]^ Significantly, ECM composition depends on aspects like tissue and tissue compartment, physiological or diseased state.^[^ ^7^, ^10^ ^]^ A special subclass of ECM is the basement membrane, which acts as a separation between epithelial or endothelial cell monolayers and connective tissue.^[^ ^11^ ^]^

One example of such a matrix is the basement membrane extract Matrigel. It has become a crucial ECM substitute in organoid research, providing the required structural scaffold for growth and differentiation.^[^ ^7^, ^12^ ^]^ Since its discovery and commercialization, it has revolutionized the field of cell biology and tissue engineering. First described in the 1980s and later named Matrigel, it has become an invaluable tool in various research applications due to its unique composition and properties.^[^ ^12^, ^13^, ^14^ ^]^ Matrigel is composed of a complex mixture of structural basement membrane proteins, growth factors, and other bioactive molecules.^[^ ^15^, ^16^, ^17^ ^]^ It mimics the microenvironment of diverse cell types and tissues, providing a 3D scaffold that supports cell growth, differentiation, and organization.^[^ ^12^ ^]^ It is a complete matrix option that is ready to use and convenient to apply. Therefore, Matrigel is a component in numerous applications, from growth substrate coating in stem cell research to 3D culture approaches like iPS‐ and patient‐derived physiological or tumor organoids for disease modeling.^[^ ^18^, ^19^, ^20^ ^]^ Additionally, it is particularly useful for embedding procedures in organoid cultivation protocols due to its ability to form a gel at physiological temperatures.

### 1.1 The Need for Alternative Matrices as Matrigel Replacements

**Figure 1:**
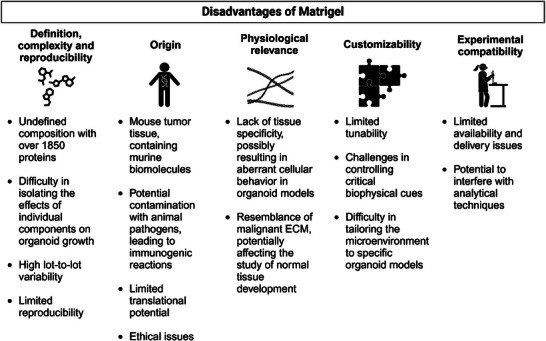
Summary of the key disadvantages of Matrigel. Details are reviewed elsewhere.^[^ ^21^ ^]^ Created in BioRender. Wolff, L. (2025) https://BioRender.com/ixbs1n3.

Despite the many advantages, Matrigel has several scientific and ethical drawbacks (extensively reviewed elsewhere^[^ ^21^ ^]^ and summarized in **Figure** **1**).

It is derived from Engelbreth–Holm–Swarm (EHS) mouse sarcoma and contains a vast mixture of basement membrane components. It includes structural proteins like laminin and collagen type 4, growth factors like epidermal growth factor (EGF) and insulin‐like growth factor (IGF‐1), and bioactive molecules like matrix metalloproteinases (MMPs), cytokines and chemokines – with variations in the specific composition between batches and potentially within lots.^[^ ^15^, ^16^, ^17^, ^22^ ^]^ More than 1850 proteins have been identified in Matrigel, making it incredibly complex.^[^ ^16^ ^]^ This high complexity and lack of standardization can introduce variability and inconsistency to experiments and challenge data comparison across studies. It also complicates distinguishing the impact of individual factors on organoid growth and development.^[^ ^16^, ^23^, ^24^ ^]^

Additionally, the animal origin of Matrigel raises scientific concerns regarding contamination and immunogenicity in human cell cultures and ethical concerns due to its production procedure (see below, subchapter “The relevance of xeno‐free matrices in human research”).

Considering the matrix source involves evaluating its physiological or pathological context. The specific composition of an ECM varies with developmental stage and tissue type, showing notable differences between healthy and diseased tissues.^[^ ^18^, ^25^ ^]^ The matrisome, which includes all structural and functional proteins and associated factors that make up the ECM, differs substantially between malignant and native states.^[^ ^25^ ^]^ In addition, the versatility of Matrigel across a range of applications results in a lack of specificity for distinct tissue models. This generalist nature can lead to deviant cellular behavior and poor representation of tissue‐specific architecture and function. For example, in a cerebellum organoid model, encapsulation with Matrigel, compared to matrix‐free organoids from the same protocol, led to aberrant lineage commitment, disrupted migration and proliferation, and increased variability in cellular composition.^[^ ^26^ ^]^ Furthermore, in a study preprint comparing the development of brain organoids encapsulated in Matrigel or in the absence of an extrinsic matrix, differences in cell patterning and axis formation were found.^[^ ^27^ ^]^ Finally, a study on gastrointestinal organoids found inherent compositional differences between decellularized gastrointestinal ECM and Matrigel; the former mainly comprised collagens and proteoglycans, while the latter contained glycoproteins.^[^ ^28^ ^]^ These examples underscore Matrigel's limitations in accurately replicating tissue‐specific architecture and function across different organoid models.

Matrigel also has limited tunability regarding matrix properties. Matrix stiffness is a particularly decisive factor influencing stem cell fate decisions and differentiation processes. For example, neural crest stem cells differentiated into smooth muscle cells when seeded on a stiff scaffold, whereas they differentiated into glial cells on a soft scaffold.^[^ ^29^ ^]^ Furthermore, mesenchymal stem cells react to matrix stiffness with lineage‐specific differentiation. They acquire a neuronal morphology on brain tissue‐like gels (soft), myoblast‐like shapes on muscle‐like scaffolds, and osteoblast‐like properties on osteoid‐like matrices (stiff).^[^ ^30^ ^]^ Additionally, a study with hepatic organoids showed that organoid formation and proliferation were optimal when physiological liver stiffness was mimicked with the hydrogel.^[^ ^31^ ^]^ Similarly, adjusting the matrix to match fibrotic liver ECM resulted in reduced organoid formation and upregulation of hepatic injury‐related genes.^[^ ^31^ ^]^

Aspects like stiffness or degradability can be adjusted to some extent via Matrigel protein concentration or by combining it with additional materials such as Collagen type 1.^[^ ^21^, ^32^, ^33^, ^34^ ^]^ However, inherent variation in composition aggravates full control.

In addition, the vast amount of biomolecules in Matrigel can influence analytical techniques like transcriptomic or proteomic profiling based on RNA isolation or mass spectrometry, making rigorous separation of sample and matrix necessary.^[^ ^35^ ^]^

Finally, more recently, practical issues such as limited availability and delivery difficulties (for example, during COVID‐related lockdowns) have challenged the conduct of large‐scale experiments.

### 1.2 The Relevance of Xeno‐Free Matrices in Human Research

Another significant drawback of Matrigel is its origin. The matrix contains murine proteins and peptides since it is extracted from mouse EHS tumor tissue.^[^ ^36^ ^]^ Especially in human research, using nonhuman components involves the risk of contamination with animal pathogens, may trigger immunogenic reactions, and complicates the translation of experimental results to clinical applications.^[^ ^21^ ^]^ Also, direct clinical and pharmaceutical use of Matrigel is hindered by regulatory and economic aspects, like Good Manufacturing Practice (GMP) conformity, clinical‐grade manufacturing, and costs.

In human research, the term “xeno‐free” indicates the absence of materials from nonhuman sources. Xeno‐free matrix options are becoming increasingly crucial as alternatives to Matrigel, particularly in cell culture and tissue engineering. Working with xeno‐free media and reagents addresses concerns regarding contamination and immunogenicity in human cell cultures. Matrices free from animal‐derived components provide a safer, more consistent platform for research and clinical applications.

Another notable concern is the application of animals and their components in models addressing research questions of human physiology, pathology, and therapy. There are substantial differences between humans and mice that might influence experimental results. On the transcriptional level, transcription factor binding in promoters of orthologous genes varies between both species despite conserved binding sequences.^[^ ^37^ ^]^ In addition, human and mouse tissues share a conserved basic overall architecture, but species‐specific gene expression patterns and cell type proportions can significantly impact functionality and translatability.^[^ ^38^, ^39^, ^40^, ^41^ ^]^ Furthermore, the human and murine immune systems differ, among others, in cellular balance, the presence of receptors, and signaling pathway components.^[^ ^42^, ^43^ ^]^ As a result, experimental outcomes may not accurately reflect human physiology and are thus not easily extrapolated to clinical settings.

To ensure the clinical relevance of research outcomes, there is a growing interest in using xeno‐free alternatives in experimental protocols. This shift enhances the translational potential of research findings and aligns with ethical and safety considerations, promoting more robust and reliable scientific investigations directly applicable to human health.

## 2 Demands on Matrix Alternatives

**Figure 2:**
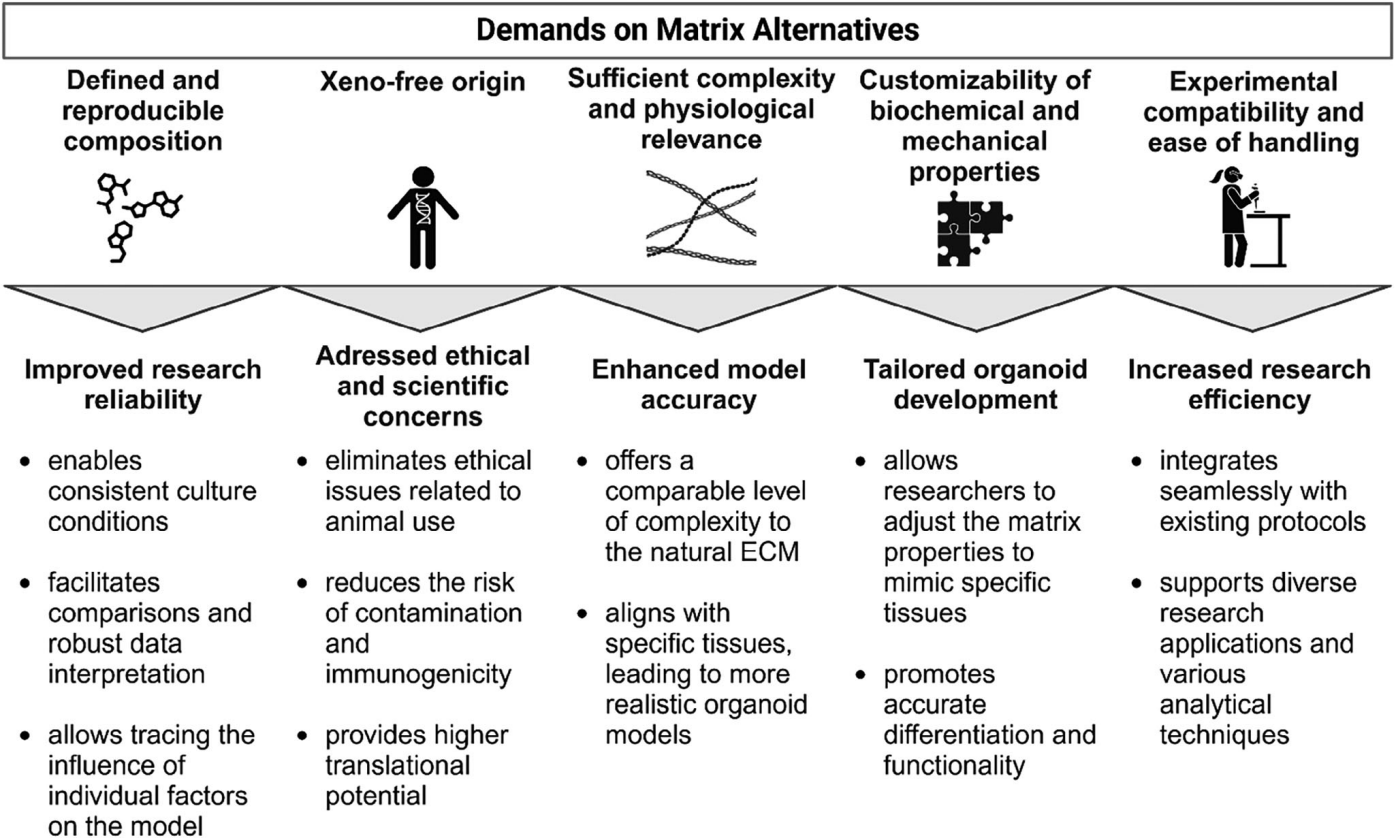
Demands on matrix alternatives and their respective implications for research and scientists, as also reviewed in Refs. [21, 23, 24] Created in BioRender. Wolff, L. (2025) https://BioRender.com/2jppnp2.

Identifying the drawbacks of Matrigel underscores the scientific demands on matrix alternatives, including biomaterials and synthetic scaffolds. Key requirements for Matrigel replacements include a defined and reproducible composition, sufficient complexity to address research questions, physiological relevance, and appropriate origin of components. Additionally, these alternatives should offer modifiability, flexibility, and ease of use (**Figure** **2**).

These aspects are essential to consider not only in matrix design from scratch but also when selecting suitable scaffolds from already available options (see the selection checklist in Tables S1 and S2, Supporting Information).

### 2.1 Defined and Reproducible Composition

Undefined matrices like Matrigel, rich in various factors, mimic the natural extracellular matrix and support cell and organoid growth. However, their versatility is offset by significant composition variability, making it challenging to reproduce experimental conditions. In contrast, precisely defined matrices improve reproducibility by offering consistent culture conditions, enabling reliable comparisons between experiments and facilitating robust data interpretations. When designing new matrices from scratch, creating scaffolds with well‐defined compositions is essential to optimize organoid systems and study the influence of specific components, such as mechanical properties, cell‐matrix interactions, and biochemical signaling. Similarly, when selecting from available matrix options, prioritizing those with clearly defined and reproducible compositions offers the same benefits – enhanced experimental consistency and improved capacity to trace the role of individual factors in organoid development. Thus, whether designing or choosing a matrix, focusing on a defined composition is critical for advancing scientific and translational applications.

### 2.2 Sufficient Complexity and Physiological Relevance

Matrigel's debatable physiological relevance due to its origin from malignant tissue and its complex composition make it nonspecific for various tissue models. This putatively leads to deviant cellular behavior and poor representation of tissue‐specific architecture and function, as described above.

Applying the same matrix to different organoid systems (protocols for Matrigel embedding cover kidney, thyroid, liver, brain, lung, intestine, prostate, breast, esophagus, gastric, ovarian, and pancreas organoids; reviewed in Refs. [21] and [24]) might adversely affect tissue development and eventually produce misleading experimental data.

Therefore, the biological, chemical, and mechanical properties of the native tissue must be carefully considered in scaffold design or when selecting from available matrix options. Matrigel alternatives are expected to offer a similar complexity while ensuring defined compositions relevant to physiological conditions. Hence, it is essential to incorporate the necessary structural and biochemical cues into the matrix (or to select a respective scaffold) to support cell adhesion, migration, and differentiation, mimicking the native microenvironment.

### 2.3 Customizability of Biochemical and Mechanical Properties

As a consequence of the previous considerations, modifiability, and customizability must be considered in matrix design or selection. Scaffold alternatives with a well‐defined composition offer high reproducibility. To ensure physiological complexity, they require the ability to incorporate factors and modify chemical properties as needed. Additionally, consistent mechanical behavior is essential, allowing researchers to mimic tissue characteristics precisely. Customization involves adjusting stiffness for differentiation cues, incorporating cleavage sites for controlled degradation, and integrating linking points for cell adhesion molecules.

The flexibility to tune the mechanical stiffness, add biochemical cues, and support cell‐matrix interactions ensures that the matrix can be adapted to mimic the complexity of different organs, providing a versatile platform for organoid culture and regenerative medicine applications.

Various studies have highlighted the significant impact of these variables on cell proliferation, differentiation, and overall organoid development.^[^ ^30^, ^44^ ^]^ Therefore, precise control of biophysical and mechanical properties is essential for tailoring the microenvironment of specific organoid models and a key requirement for an optimal matrix candidate.

### 2.4 Compatibility with Experimental Settings and Ease of Handling

Matrigel is widely used in many research applications, from 2D cell culture to advanced tissue engineering and organoid studies.^[^ ^21^ ^]^ Its biological compatibility with various cell types, supporting their growth and differentiation, is crucial for diverse research studies. Consequently, any alternative matrix is expected to match this versatility and seamlessly integrate into existing experimental protocols. However, a significant advancement would be the development of scaffolds tailored to specific models or a model‐specific matrix selection, offering properties optimized for particular experimental needs. Alternatives must also support long‐term culture, maintaining organoid viability and functionality over extended periods. Furthermore, technical compatibility is essential, ensuring that the matrix supports multiple analytical techniques, such as imaging, molecular assays, and high‐throughput screening, enabling researchers to obtain comprehensive data while employing these alternatives.

### 2.5 Xeno‐Free Origin

As pointed out above, the murine origin of Matrigel raises two significant concerns. Ethically, the production and propagation procedure is questionable: mice carry tumors that amount to 20–25% of their weight.^[^ ^36^ ^]^ Scientifically, incorporating animal‐derived components in human research poses risks such as contamination, immunogenicity, and limitations in translatability. To address these concerns, researchers increasingly turn to xeno‐free alternatives, which can be sourced from human cells or tissues, or developed entirely synthetically. Still, regulatory barriers, including GMP‐compliant and clinical‐grade production, and economic aspects, like cost and scalability, must be considered.

## 3 The Gold Standard Status of Matrigel

Despite its disadvantages and the availability of alternative scaffold options, Matrigel remains extensively used in organoid culture. It is considered the gold standard for several reasons. First, it has a long‐established and substantial body of literature supporting its use, with more than 14 000 results in PubMed searches using the keyword “Matrigel.” Its established protocols and experimental workflows make it convenient for researchers familiar with its application. Moreover, there is reluctance among scientists to switch to alternatives without robust evidence of their compatibility and effectiveness in organoid culture. Second, Matrigel has a complex composition resembling the general growth environment for several cell types. While synthetic matrix substitutes excel in standardization and customization, they may not fully capture the complexity and biological cues of a complete ECM. Expectations toward alternative scaffolds are high, and Matrigel is set as the standard regarding complexity. Third, transitioning to substitute matrices requires significant optimization and evaluation efforts, as each scaffold has unique properties, and every organoid system has specific requirements. Considering and acknowledging these requirements beforehand and collating them with available matrix options is complex, and the following assessment and optimization process is tedious and costly. Additionally, these experiments require a reference for comparison – which is typically Matrigel.

Overall, while alternative matrices show promise, achieving widespread adoption of Matrigel replacements requires further research, standardization, and validation to meet the specific needs of organoid culture systems.

## 4 The Rocky Road of Implementing Alternative Matrices

While the need for alternatives to Matrigel has long been acknowledged, no matrix has yet achieved similar widespread popularity and applicability. Efforts are ongoing to develop and validate novel matrix options and select and assess already available substitutes in diverse applications. However, several interconnected challenges complicate the pursuit and implementation of Matrigel substitutes.

### 4.1 Challenge 1: The Expectations Toward New Matrix Alternatives Are High

As already summarized above, there is a demand for matrices that replicate all the beneficial properties of Matrigel while addressing its disadvantages—such as being ready‐to‐use and xeno‐free, well‐defined yet complex. These expectations drive innovation but also hinder progress by setting impractically high standards.

### 4.2 Challenge 2: The Development of New Scaffolds Is not Trivial

Creating new matrices meeting high standards by combining properties such as biocompatibility, tunable stiffness, and 3D applicability is not trivial. It is complex and requires interdisciplinary expertise spanning chemistry, material sciences, biology, and medicine. Purely chemical considerations may yield matrices that have adjustable mechanical properties but do not work in vitro or in vivo, because polymerization or cross‐linking occurs under conditions incompatible with biological systems or working under sterile conditions. Currently, synthetic or natural biomaterial scaffolds are predominantly developed by labs specializing in materials science and bioengineering, while organoid researchers often rely on collaborations with these labs or commercially available options.^[^ ^28^, ^45^, ^46^ ^]^ However, the scarcity of functionalized, ready‐to‐use matrices capable of forming 3D scaffolds poses a significant challenge.

### 4.3 Challenge 3: Selecting a Suitable Matrix from the Available Options Requires Careful Considerations and Compromising

As a result of the previous challenges, researchers face the task of selecting the most appropriate matrix alternative from the pool of existing commercially available options. The prerequisites of the experimental and study design must be identified and carefully compared against the properties of potential replacement candidates. Additionally, the applicability of matrices must be tested for each specific model and application, resulting in a potentially tedious and expensive process. This could collide with the economic and resource constraints faced by many labs.

### 4.4 Challenge 4: Clear Assessment Criteria Regarding Applicability and Biocompatibility Are Not Established

Many suppliers of matrix alternatives and labs developing their own matrices provide information on their properties. Apart from gelation kinetics, these data mainly include biomaterial science criteria like stiffness, elasticity, pore size, or purity. However, these details are often only available upon request and do not necessarily provide information about compatibility with cellular systems or applicability for specific handling procedures. For some scaffolds, experiences with selected organoid systems are shared, but the compatibility with specific models has to be assessed on a case‐by‐case basis.

### 4.5 Challenge 5: Publication Bias Undermines the Fair Assessment of Alternative Matrices

Matrigel has an impressive presence in the literature, with a record exceeding 14 000 publications (**Table** **1**). In contrast, the more recent hydrogel product VitroGel (15 hits) is the most popular xeno‐free whole‐matrix alternative. Apart from this purely quantitative aspect, publication bias can further hinder the implementation of new scaffolds. It favors studies that report positive results, probably leading to an overrepresentation of favorable outcomes for Matrigel in a direct and undifferentiated comparison with alternative matrices. This can falsely suggest that Matrigel is superior to novel scaffolds, even when the evidence is inconclusive. Moreover, researchers tend to cite studies supporting their preferred matrix, perpetuating bias against novel options. Finally, many studies comparing Matrigel to putative substitutes have small sample sizes, making it difficult to draw firm conclusions about the relative efficacy of the two materials. Also, different experimental standards and conditions prevent comparisons between studies.^[^ ^47^ ^]^ This can make it difficult for researchers to justify switching from Matrigel to another scaffold, even if the novel matrix has some potential advantages.

**Table 1:** Selected commercially available matrix options and their properties compared to Matrigel.

Product	Details	Origin	Complexity	Customization	Application^a)^	References in Pubmed^b)^
Matrigel and equivalents						
Matrigel	EHS tumor extract	murine	high	limited	2D + 3D	14 271
Cultrex Ultimatrix						37
ECM‐Gel						92
Whole matrix alternatives						
MaxGel	cell culture extract	human	high	limited	2D	7
HumaMatrix	tissue extract				2D + 3D	0
Vitrogel Organoid	polysaccharide‐based	synthetic	limited	possible	3D	15
TrueGel 3D	dextran or PVA backbone					0
Single ECM components						
PureCol	skin extract	bovine	limited	limited	2D + 3D	6
VitroCol	cell culture extract	human				0
HumaDerm	skin extract					2
HyStem	hyaluronic acid‐based	synthetic		possible		31

^a)^ 2D: two‐dimensional models; 3D: three‐dimensional models;

^b)^ A simple one‐term search for the product name determined the number of references in Pubmed; ECM: extracellular matrix; EHS: Engelbreth–Holm–Swarm; PVA: polyvinyl alcohol.

The combination of these interacting challenges contributes to a situation where alternative matrices are not given a fair chance to compete with Matrigel, even if they have the potential to be more effective or suitable.

## 5 Navigating Challenges in the Transition to Matrigel Substitutes

Overcoming the multiple challenges in developing, validating, assessing, and implementing novel matrix candidates requires a multifaceted approach.

### 5.1 Exploring Alternatives to Matrigel – From a One‐for‐All Solution to a Tissue‐ and Model‐Specific Approach

As discussed previously, the expectations toward potential Matrigel substitutes are high. Novel matrix candidates should preferably unite Matrigel's favorable properties while avoiding its drawbacks. Thus, a shift toward seeking tissue‐ and model‐specific alternatives appears feasible. This does not only affect the design of novel matrix scaffolds but also the process of selecting the most suitable matrix candidate from the options already available. The latter entails acknowledging and considering the requirements of the respective organoid model, study purpose and lab resources and collating them with the properties of the available matrix options (a selection checklist and matrix evaluation tool are provided in Figure S1 and Tables S1 and S2, Supporting Information).

The limitations of Matrigel (and equivalent basement membrane formulations available from other companies, such as Cultrex Ultimatrix (biotechne) or ECM‐Gel (BioReagent) (Table 1)) have prompted researchers to find alternative scaffolds.

In recent years, there has been an active exploration of matrices that meet the scientific and practical demands associated with organoid culture systems offering more suitable growth environments.^[^ ^21^, ^48^, ^49^, ^50^, ^51^, ^52^, ^53^ ^]^ These innovations include natural and synthetic matrices with varying levels of definition and complexity, ranging from single ECM components to whole‐matrix solutions. We summarize selected commercially available options in Table 1, comparing informative scientific and technical criteria, like structural and compositional features. Due to a lack of standardized peer‐reviewed data on these products, we rely on manufacturers’ claims and datasheet information, which should be interpreted cautiously, especially when considering applications beyond the manufacturers’ specifications, underlining once more the need for standardized benchmark analyses.

#### 5.1.1 Biological and Synthetic Whole‐Matrix Alternatives

These matrix candidates are either sourced from cells or tissues or based on entirely synthetic polymers. Tissue extracts present an innovative alternative to Matrigel as the tissue source can be selected according to the organoid model of choice. The process of tissue decellularization removes cellular components from biological tissues while preserving the extracellular matrix structure and composition. Thus, the bioactive molecules and signaling cues in the physiological ECM are retained, providing the relevant native environment of the target tissue. For example, Kim and coworkers utilized ECM extracted from pig stomach and intestine to culture human intestinal organoids, comparing its composition and utility with Matrigel.^[^ ^28^ ^]^

Mainly, specialized research groups follow this approach, but commercial options for specific tissues are also available.^[^ ^28^, ^46^ ^]^ For instance, MaxGel ECM Hydrogel is produced from the in vitro co‐culture of human fibroblasts and human epithelial cells. However, this product is primarily used for coating plasticware rather than forming stable 3D gels or domes encapsulating organoids.^[^ ^54^ ^]^ Another strategy is approached by HumaBiologics, a company offering fully biological matrices extracted from a range of human tissues.^[^ ^46^ ^]^ One such product, HumaMatrix, is suitable for general 3D culture and can be modified to a certain degree. Limitations of this strategy entail the limited availability of suitable donor tissue, potentially challenging large‐scale application. In addition, decellularized tissue and tissue extracts underlie biological variation, leading to batch‐to‐batch variability that parallels the issues seen with Matrigel. The resulting ECM material is not per se xeno‐free but depends on the choice of tissue source. Also, the respective methodologies are not standard in every lab, and incomplete decellularization may result in residual cellular components as potential immune triggers.

In addition to naturally sourced matrices with a certain inherent biological variability, synthetic options are available. These synthetic hydrogels are 3D networks of hydrophilic but insoluble polymers (such as polysaccharides or polyvinyl alcohols) that can absorb and retain a significant amount of fluid. They can be classified as defined matrices. They offer more precise control of biological (e.g., growth factor content) and mechanical properties (e.g., matrix stiffness) through composition adjustments or cross‐linking techniques.^[^ ^55^, ^56^ ^]^ Crosslinking techniques can be physical or chemical, the former including ionic interactions (e.g., in alginate‐based hydrogels) or thermogelation (e.g., polyvinyl alcohol‐based gels), the latter comprising photopolymerization (e.g., methacrylated collagen) or click chemistry (e.g., thiolated hyaluronic acid with polyethylene glycol macromers).^[^ ^55^ ^]^

Their synthetic nature eliminates concerns regarding animal origin, potential contamination, and immunogenicity. However, they often require further functionalization to enhance bioactivity and complexity, which is achieved by incorporating cell‐adhesive motifs (e.g., peptide sequence RGD), protease recognition sites (e.g., peptide sequence GPQGIWGQ), growth factors, and ECM components.^[^ ^51^, ^57^ ^]^ VitroGel (TheWell Biosciences) exemplifies a fully synthetic but already functionalized matrix, featuring multiple formulations with varying bio‐functional ligands, mechanical strengths, and degradability. TrueGel3D Hydrogel is another example, consisting of a polymer, a thiolated crosslinker, and optional cell‐interactive reagents.

#### 5.1.2 Biological and Synthetic Single ECM Components

Apart from providing a complete ECM as a growth environment for the specific organoid model, a more minimalistic approach using individual ECM constituents is conceivable.

Biologically sourced single ECM components such as collagens, laminins, or vitronectin are commercially available. These components are favored for their biocompatibility and defined compositions. However, not all of them can form stable 3D gel structures; instead, they are primarily used as coating agents, such as laminins or vitronectin. Collagen solutions derived from bovine or human origin can be obtained with high purity, consistency, and reproducibility. Products like PureCol (bovine) and Vitrocol (human) or HumaDerm (human) are applicable for coating surfaces and forming 3D gels, depending on the supplier's specifications.

Additionally, a specifically tailored synthetic matrix can be considered an option in replacing Matrigel. Ethical concerns can be set aside, and the composition can be highly reproducible and defined. One example is HyStem (Advanced BioMatrix), which consists of a hyaluronic acid (HA) component that is further chemically modified with thiol groups, supplemented with the linking agent polyethylene glycol (PEG) diacrylate. We have classified this naturally occurring polymer as synthetic, as it is not extracted from tissue but of recombinant microbial origin, with subsequent chemical modification. Additionally, the polymerization process is initiated by adding a synthetic crosslinker. This xeno‐free matrix is customizable, providing an alternative for cell types native to HA‐rich environments.

In synthetic alternatives, properties like stiffness or degradability can be generally adapted to the specifications of the target tissue, offering flexibility. However, this high degree of tunability and full control over composition is double‐edged. It provides high flexibility but also demands particular lab equipment and a thorough understanding of material properties, biological systems, and their interactions. It is no ready‐to‐use solution and depending on the intended application, additional functionalization of the matrix scaffold is necessary. Accordingly, extensive characterization and evaluation can potentially become time‐consuming and costly.

**Figure 3:**
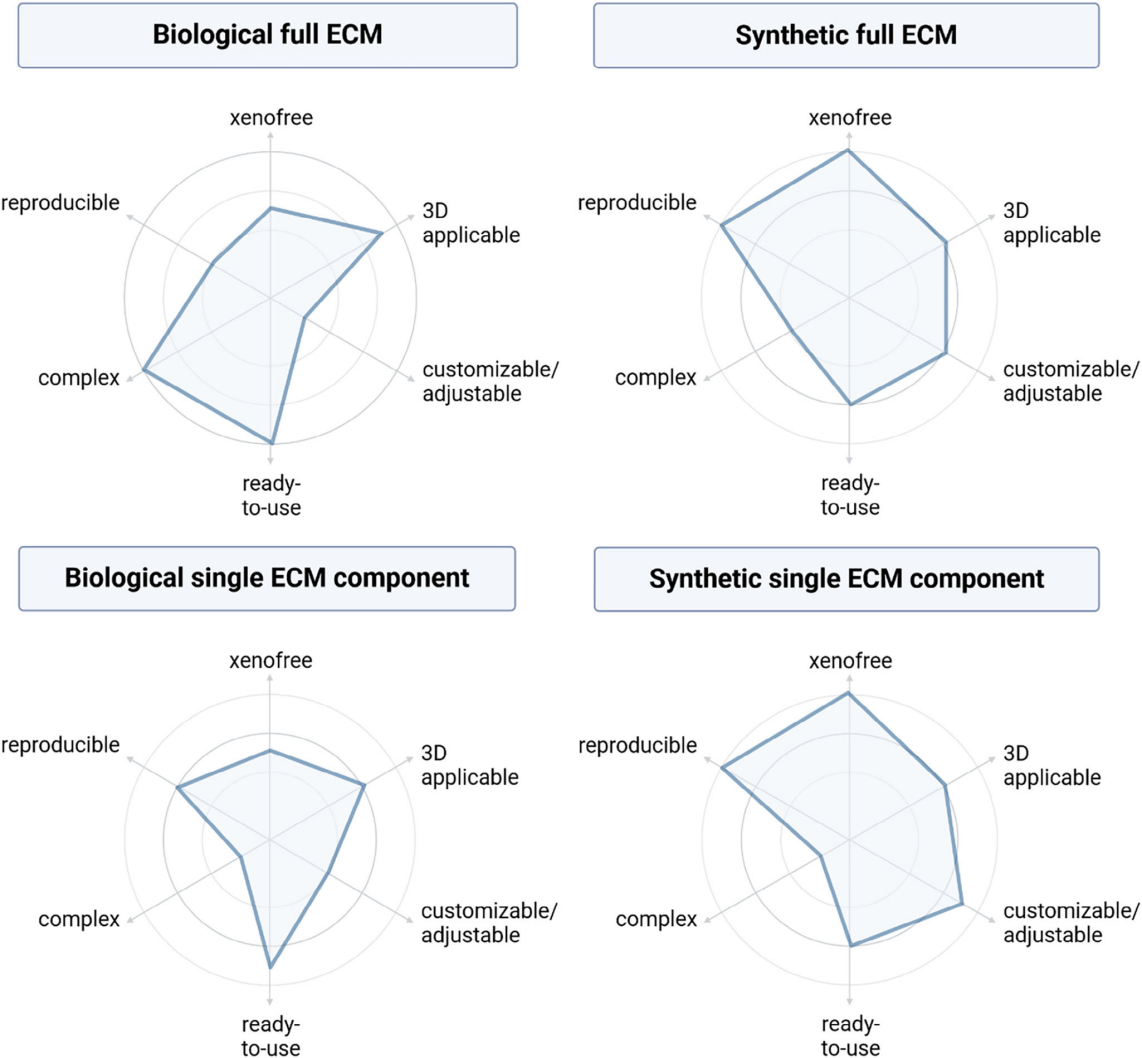
Organoid SCaffold Assessment Radar (OSCAR), illustrating the comparison of key matrix properties important for scaffold selection; grouped by main scaffold classes. Created in BioRender. Wolff, L. (2025) https://BioRender.com/fr5680y.

The decision for one or the other matrix approach depends strongly on the focus of the study and the model used, and compromises must be considered (**Figure** **3**; more aspects for consideration regarding matrix selection are summarized in the matrix selection checklist Tables S1 and S2, Supporting Information).

### 5.2 Introduction of Standardized Validation Parameters and Quantitative Assessment Criteria

Designing, developing, and validating alternative scaffolds is not trivial and requires a collaborative approach that combines expertise from chemistry, materials science, biology, engineering, and medicine. In previous years, several matrix substitutes have been made commercially available (examples in Table 1), enabling researchers from different fields to explore alternatives for their research. To collate the requirements of the organoid model of choice with the properties of the available matrix options, data on these properties must be accessible. Companies mainly provide material‐focused information together with application notes. This includes the matrix source and main components (biological products) or molecules (synthetic products), mechanical strength and gelling kinetics, the pore size of the polymerized gel, and dome formation ability. Further details can often be provided upon request. However, data on biological compatibility is not always present, and there are no standardized criteria or test pipelines. Hence, assessing the compatibility of the matrix with a specific organoid model remains the responsibility of the respective researchers. To facilitate this process, we suggest assessment criteria to evaluate the suitability of different matrix candidates for the particular model of choice.

#### 5.2.1 Dome‐ or Shell‐Forming Ability and Stability of the Matrix

Some alternatives to Matrigel are ideal replacements for surface coating procedures, e.g., human recombinant vitronectin as a growth substrate for induced pluripotent stem cell culture.^[^ ^58^ ^]^ However, most of the single ECM components and some commercial tissue‐specific extracts are not applicable to form 3D gels. Apart from sandwich‐like techniques used in specific protocols, e.g., for lung or intestinal organoids, many other applications in 3D culture require matrix alternatives that form a stable dome or shell surrounding the organoids or their precursors.^[^ ^5^, ^59^, ^60^ ^]^ This property can be evaluated together with the stability during normal cell culture handling, like media change, and stability throughout the experimental timeline.

#### 5.2.2 Embedding Rate and Detachment Factor (Where Applicable)

**Figure 4:**
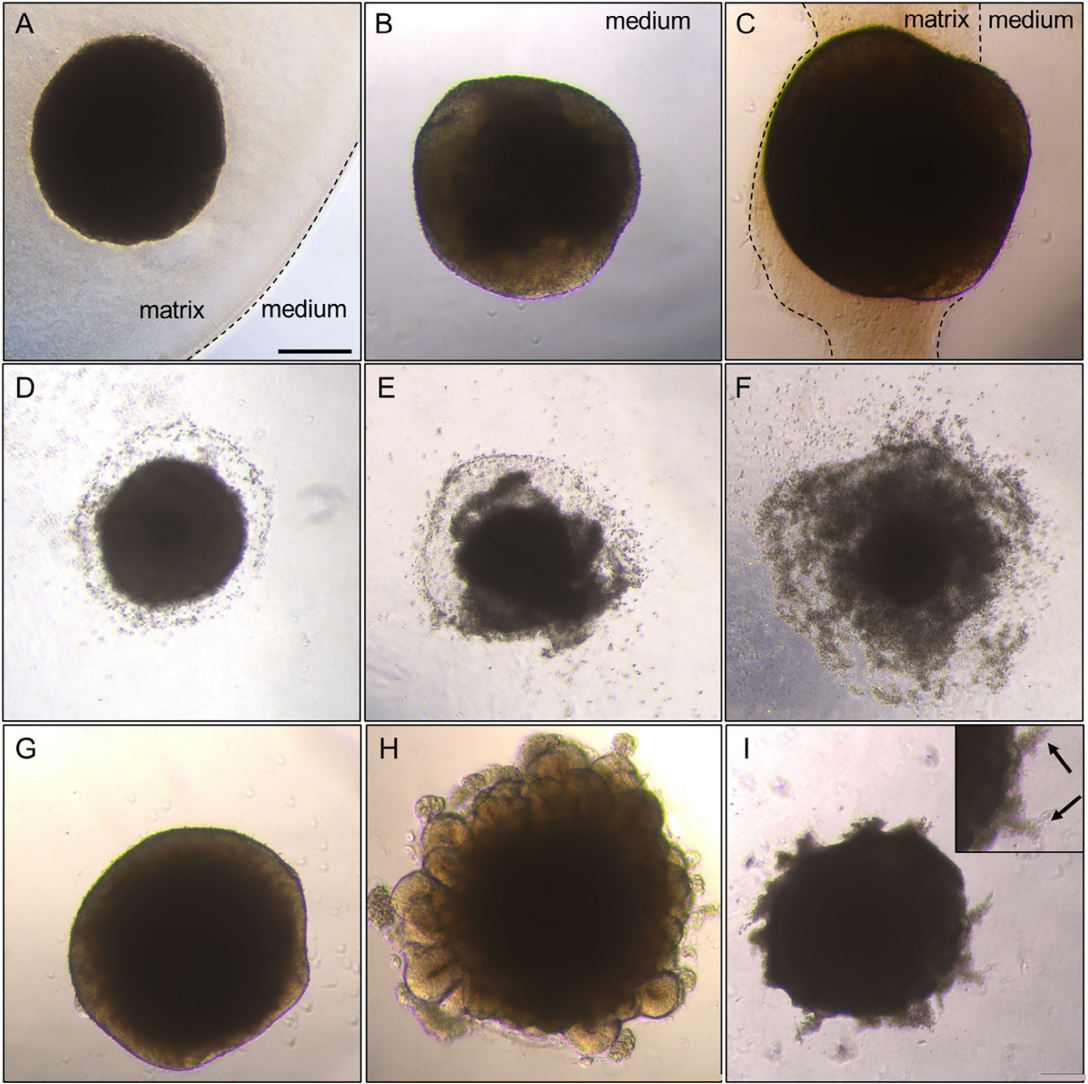
Examples of different matrix embedding results of cerebral organoids at d14, disintegration states of embedded embryoid bodies (EB) in different matrices at d8 (= d1 after embedding), and differentiation statuses of similar organoids at d15 (Lancaster protocol ^[^ ^5^ ^]^). A) The organoid is fully embedded and surrounded by the scaffold. B) The organoid is fully detached from the matrix after the embedding procedure. C) The organoid remains partially attached to the scaffold, with regions directly exposed to the surrounding culture medium. D) The borders of the EB are uneven, and single cells are shed, conforming with a beginning disintegration. E) The EB sheds single cells and tissue fragments, resulting in partial disintegration. F) The EB dissociates into fragments and single cells, corresponding to an advanced disintegration. G) No external matrix: the translucent, smooth borders, in contrast to the dense core of the organoid, indicate the initiated formation of neuroepithelium. H) External matrix: emerging from a dense core, a bright budding border area emerges, corresponding to expanding neuroepithelium. I) External matrix: the density of the organoid is uniform, and extensions start to grow into the surrounding matrix. Size bar 250 µm.

The procedure of embedding organoids in Matrigel is well‐established and considers its specific polymerization behavior and properties. The embedded organoids (either dome, shell or sandwich technique) interact with the matrix, grow into it, and consume it until the remaining matrix detaches or is entirely consumed. While direct quantitative measurement of matrix consumption is not typically feasible with current protocols, it can be qualitatively assessed through routine monitoring of organoid development. Successful matrix remodeling and integration can typically be observed by growth and tissue expansion into the matrix over time, often accompanied by a gradual replacement of matrix with newly formed tissue. In contrast, if the matrix is not consumed but rather detached, it typically floats in the surrounding medium, indicating a lack of integration. For dome or shell formation protocols, the embedding procedure using alternative scaffolds will depend on their respective polymerization mode and may require optimization (**Figure** **4**A–C).

Here, we introduce the embedding rate as a quantitative indicator of the success of the embedding procedure. Although it does not directly predict functional or structural organoid development, the embedding rate serves as a practical tool to evaluate the technical compatibility of different matrices with particular protocols in terms of handling and methodological feasibility. The embedding rate is defined by the fraction of organoids that remain embedded in the matrix until the end of the experiment or until the matrix is fully consumed. The inversely proportional detachment factor summarizes the fraction of organoids that have detached from the scaffold precociously. We propose ≥75% as a reasonable operational benchmark based on our experience. Regarding statistical power analysis and sample size calculations, a 75% threshold would mean a 25% dropout rate for statistical analysis. Sample size calculations refer to the number of data points required for statistical analysis, not the initial number of samples to be prepared. As a result, appropriate starting sample sizes would be 25% higher than the actual computed sample size. Therefore, we consider an embedding rate of ≥75% an acceptable threshold, reflecting a pragmatic compromise between technical feasibility, resource constraints, and reproducibility needs. By comparing the embedding or detachment rates across different scaffolds tested in a single study, conclusions can be drawn about the embedding process and the practical suitability of each matrix for the specific model. As a result, embedding procedures and possibly gelation processes can be quantified, optimized, and adapted to the respective procedure.

#### 5.2.3 Integrity Rate to Evaluate Tissue Integrity and Disintegration

Another property of the alternative matrix concerns its biocompatibility and whether its composition supports or interferes with organoid growth. Possible scenarios range from the migration of cells out of the main body to its complete dissolution, resulting in the partial or total disintegration of the embedded organoid (Figure 4D–F). Here, we introduce the integrity rate as the ratio of intact to disintegrated organoids calculated for each scaffold, providing insights into how many intact organoids are maintained until the end of the culture period.

By comparing the integrity rates of different scaffolds in a study, conclusions about the biocompatibility and suitability of the matrices for a specific model can be drawn. Again, we consider a threshold of ≥ 75% as an acceptable rate.

#### 5.2.4 Analysis of Size and Differentiation of Embedded Organoids

To evaluate whether the surrounding matrix contributes to the development of the organoid, the degree of differentiation can be assessed. In cerebral organoids, the presence and growth of expanding neuroepithelium exhibited by budding of the embedded EBs can be easily observed by light microscopy and compared between different matrix alternatives (Figure 4G–I).

Additionally, a growth rate can be established throughout each experiment. Differences in observed size and differentiation depend on the presence and amount of growth factors and other biomolecules in the matrix and surrounding medium. If necessary, additional external factors can be added to the system, and their effect can be assessed using these measurements. These measures can be obtained as a rapid initial indication of matrix compatibility. However, in later stages of organoid validation, functional assays (e.g., electrophysiological measurements of brain or heart tissue organoids using patch clamp or microelectrode array) and the detection of specific markers (e.g., SOX2 for neural progenitors and NeuN for postmitotic neurons in cerebral organoids) are essential for a complete study of differentiation and development.

Integrating biomaterial data, applying quantitative model‐specific evaluations (**Table** **2**) using standardized criteria, and adopting a more tailored, tissue‐specific approach could lead to the successful implementation of alternatives to Matrigel. This approach can comprise whole‐matrix products and single ECM components, either biologically sourced or synthetically generated.

**Table 2:** Summary of the criteria applicable for matrix evaluation.

Criterion	Details
Practical applicability	The matrix is applicable for free‐floating culture using shell‐embedding.
	The matrix is applicable for static culture using a dome or sandwich formation.
	The matrix is sufficiently stable for everyday cell culture handling.
Embedding rate	Threshold of 75%.
	The matrix interacts accordingly with the organoid or precursor to remain attached until consumption or the end of the experiment.
Integrity rate	Threshold of 75%.
	The matrix is biocompatible with the organoid or precursor and supports its well‐being and growth.
	The matrix is not toxic or inhibitory for the organoid or precursor.
Analysis of size and differentiation	The matrix supports the growth and differentiation of the organoid or precursor.

## 6 Conclusions

Organoids are self‐organizing miniature organ models derived from stem cells or tissue‐specific progenitor cells, offering a promising tool for studying tissue development and disease modeling. Matrigel has become integral to organoid research as an external growth scaffold, but it has several drawbacks, including reduced reproducibility and translatability (Figure 1). The resulting demand for alternatives is accompanied by high expectations toward novel scaffolds (Figure 2). Although several alternative options are already available (Table 1), Matrigel remains the gold standard.

Several challenges complicate the implementation of Matrigel substitutes. The expectations for matrix alternatives are high, the development of novel scaffolds is not trivial, and selecting the appropriate matrix from available commercial options can be difficult. Furthermore, no clear criteria for assessing their applicability and compatibility have been established. Finally, publication bias can skew the competition between the traditional matrix candidates and novel options.

To address these challenges, a combined approach seems necessary. First, when exploring alternatives to Matrigel, a model‐ and tissue‐specific approach should be preferred to a one‐for‐all solution. Second, standardized validation parameters and quantitative assessment criteria should be introduced. This includes making as much data on alternative scaffolds available to researchers as possible to facilitate the selection of a suitable matrix for their research application. Here, we provide an organoid scaffold assessment radar (OSCAR; Figure 3) and a matrix selection checklist (Tables S1 and S2, Supporting Information) to facilitate selecting a matrix from available options and suggest criteria for the in‐house validation regarding matrix applicability for the intended purpose.

Introducing practical and, in part, quantitative assessment criteria, as suggested in this review, such as matrix stability and embedding rate, integrity rate, and differentiation potential (Figure 4 and Table 2), helps to assess the applicability of the matrix for a specific organoid model. Replacing the idea of a one‐for‐all solution with a more tissue‐ and model‐specific strategy would further increase the awareness of particular matrix properties and tissue requirements. Finally, leaving overly high expectations toward new scaffolds behind and keeping in mind the limitations of Matrigel will facilitate the implementation of novel matrix options.

## Conflict of Interest

The authors declare no conflict of interest.

## Supporting information



Supporting Information
