# Supplementary material for: Rethinking Matrigel: The Complex Journey to Matrix Alternatives in Organoid Culture
Source: Adv Sci (Weinh). 2025 Nov 7;12(47):e08734. doi: 10.1002/advs.202508734 (PMC12713094; doi:10.1002/advs.202508734)
Supplement: Supplementary file 1 — Supporting Information [file ADVS-12-e08734-s001.docx]

Supporting Information

**Rethinking Matrigel: The Complex Journey to Matrix Alternatives in Organoid Culture**

*Lisa Wolff and Sven Hendrix**

**OSCAR template**


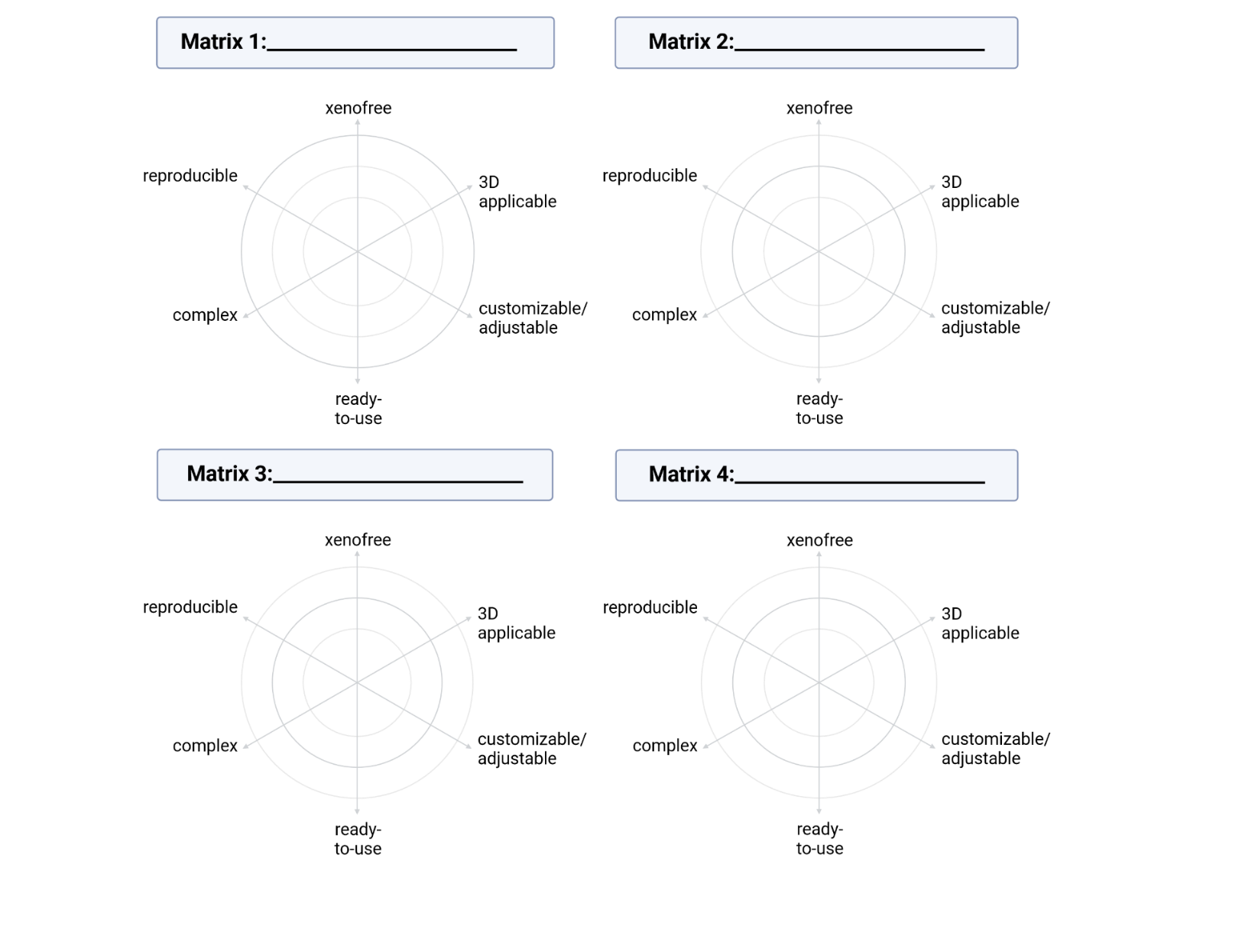


**Figure S1:** Organoid SCaffold Assessment Radar (OSCAR) template to compare the main properties of putative matrix candidates for a prospective study. Created in BioRender. Wolff, L. (2025) https://BioRender.com/w5i86l1

**Matrix selection checklist**

**STEP 1 - Define your requirements**

**Table S1:** Checklist questions to help define the requirements for a scaffold for a particular application and project.

| **Category** | **Question** | **Examples** |
| --- | --- | --- |
| **Organoid Model** | Which organoid model are you working with? | - Cerebral organoids embedded in a matrix droplet |
|  |  | - Lung or intestinal organoids using a sandwich technique |
|  | Does your cell type(s) require a particular growth environment? | - Tissue of origin’s ECM as the main component |
|  |  | - Selected growth factors critical for the model |
|  | Does your protocol require a particular matrix property? | - Embedding requiring dome formation |
|  |  | - Steps needing particular polymerization temperatures |
|  |  | - Matrix free from pro-inflammatory or unknown bioactive molecules |
|  | What is your required culture time? | - Long-term stable matrix for extended culture periods |
|  | Which matrix property is most important to be adjustable in your model? | - Modification of matrix stiffness as a developmental trigger |
|  |  | - Degradability for cell migration |
|  | What is the main purpose of the external matrix in your model? | - Structural support |
|  |  | - Delivery of growth factors or bioactive molecules |
|  |  | - Object mobilization or immobilization |
|  | What is your priority? | - Xeno-free status |
|  |  | - Defined composition or complexity |
| **Study Purpose** | Is the main purpose of the study to examine the influence of individual factors on your model system? | - Effect of presence or absence of growth factor X on organoid differentiation |
|  |  | - Retention or availability of added factors in the matrix |
|  | Is translatability or clinical application a critical aspect of your study? | - Xenofree setup for human research |
|  | Do you require an injectable matrix for your study? | - Compatibility of the matrix with injection procedures |
|  | Do you plan on bioprinting in your study? | - Matrix compatibility with bioprinting processes |
|  | Which downstream analyses are planned for the study? | - Compatibility with imaging |
|  |  | - Avoidance of interference with proteomic analyses |
| **Lab Resources** | Can you analyze matrix-related property adjustments with the appropriate devices? | - Availability of tools to measure adjustable stiffness |
|  | Can you afford detailed matrix and model assessment experiments? | - Resource needs for pre-studies (time, analyses, reagents) |
|  | Are you equipped to prepare your own matrix? | - Capability for decellularization of tissue |
|  |  | - Availability of required tissue |

**STEP 2: Evaluate the properties of available matrix options to make your decision**

**Table S2:** Summary of the general advantages and disadvantages of biological and synthetic whole matrix options or single ECM components.

| **Whole Matrix Biological** | | **Whole Matrix Synthetic** | | **Single ECM Biological** | | **Single ECM Synthetic** | |
| --- | --- | --- | --- | --- | --- | --- | --- |
| **PROS** | **CONS** | **PROS** | **CONS** | **PROS** | **CONS** | **PROS** | **CONS** |
| Retains bioactive molecules and signaling cues, providing a native-like environment | Potential presence of immune triggers from incomplete decellularization | Defined composition | May require additional functionalization to enhance bioactivity and mimic complexity | Defined composition | Potentially requires combination with other molecules for sufficient complexity | Defined, reproducible composition | Functionalization is necessary to replicate the complexity of natural matrices |
|  | May contain non-tissue-specific factors | Allows fine-tuning and customization | Demands extensive characterization and optimization, which can be time-intensive | High purity and consistency |  | Allows fine-tuning and customization | Requires thorough understanding of material properties and biological interactions |
| Can be sourced from target tissues to match particular organoid models | Potentially limited availability of suitable donor tissues for large-scale applications | High reproducibility |  | high biocompatibility due to natural origin | Some components are limited to surface coating, not forming 3D scaffolds |  | Time-consuming and costly to customize and evaluate for selected applications |
|  | Not per se xeno-free, depending on the tissue source | xeno-free | synthetic origin may affect biocompatibility |  | Not per se xeno-free, depending on the cell/tissue source |  | Complex customization processes increase experimental costs |
| ready-to-use | one-for-all solution | often ready-to-use | May require finetuning for particular models |  |  |  | Some components are limited to surface coating, not forming 3D scaffolds |
| high biocompatibility due to natural origin | Batch variability possible due to biological sourcing, affecting reproducibility |  |  |  |  | xeno-free | synthetic origin may affect biocompatibility |
